# Diffuse Melanosis and Ascites due to Metastatic Malignant Melanoma

**DOI:** 10.1100/tsw.2008.78

**Published:** 2008-05-23

**Authors:** Elena Sendagorta, Angel Pizarro, Marta Feito, Matias Mayor, Paloma Ramírez, Uxua Floristán, Rosa Feltes

**Affiliations:** Dermatology Department, University Hospital La Paz, Madrid, Spain

**Keywords:** Melanosis, melanoderma, melanuria, melanoma

## Abstract

We present a female patient who developed mucosal and skin hyperpigmentation due to metastatic malignant melanoma. Diffuse cutaneus melanosis is a rare entity that complicates a small percentage of metastatic melanomas, confering a fatal prognosis. We discuss briefly the current evidence on pathogenesis of melanosis arising from metastatic melanoma.

## INTRODUCTION

A 67-year-old, white Spanish woman presented to our office with a dark pigmented lesion on the right buttock suggestive of melanoma. Excisional biopsy confirmed our diagnosis with Clark level V, tumor thickness 6.26 according to Breslow. CT scan revealed the presence of multiple hepatic space-occupying lesions, confirmed as metastases by fine-needle aspiration biopsy. She started chemotherapy with Dacarbacine, but 4 months later, developed mucosal and skin hyperpigmentation, darkened urine, ascites, hepatosplenomegaly, weight loss of 6 kg, and asthenia. Dermatologic exam showed diffuse hyperpigmentation (Fig. 1) with a slate blue-hue (compare to the patient's skin 5 years before melanoma diagnosis [Fig. 2]), blue-tinged nails (Fig. 3), darkened conjunctivae (Fig. 4), ascites (Fig. 5) and many black-bluish papules and nodules on her skin. The urine was dark, consistent with melanuria. The patient finally died.

## COMMENT

Although diffuse cutaneous melanosis arising from metastatic melanoma is well documented[1], its incidence is low in comparison to the number of patients with metastatic melanoma. The pathogenesis is currently controversial[2,3], although Bohm et al. propose that cutaneus diffuse melanosis is linked to an excessive production of distinct humoral factors[3]. Excessive production of alpha-melanocyte stimulating hormone (αMSH) (from the tumor) in combination with hepatocyte growth factor (HGF) and endothelin-1 (ET-1) (released from distinct site of metastasis) would induce a synergistic response of normal and malignant melanocytes resulting in enhanced proliferation, melanogenesis, and motility. Excessive melanogenesis by epidermal melanocytes and melanoma cells would then be followed by a pigment incontinence, possibly forced by ET-1-mediated apoptosis of the tumor cells[3].

## References

1. Hofmann, M., Kiecker, F., Audring, H., Grefer, K., Sterry, W., and Trefzer, U. (2004) Diffuse melanosis cutis in disseminated malignant melanoma. *Dermatology* **4**, 350–352.
2. Steiner, A., Rappersberger, K., Groh, V., and Pehamberger, H. (1991) Diffuse melanosis in metastatic malignant melanoma**.** *J. Am. Acad. Dermatol*. **4**, 625–628.
3. Böhm, M., Schiller, M., Nashan, D., Stadler, R., Luger, T.A., and Metze, D. (2001) Diffuse melanosis arising from metastatic melanoma: pathogenetic function of elevated melanocyte peptide growth factors. *J. Am. Acad. Dermatol*. **5,** 747–754.
